# Assessment of Blood-Brain Barrier Function and the Neuroinflammatory Response in the Rat Brain by Using Cerebral Open Flow Microperfusion (cOFM)

**DOI:** 10.1371/journal.pone.0098143

**Published:** 2014-05-22

**Authors:** Arijit Ghosh, Thomas Birngruber, Wolfgang Sattler, Thomas Kroath, Maria Ratzer, Frank Sinner, Thomas R. Pieber

**Affiliations:** 1 Division of Endocrinology and Metabolism, Medical University of Graz, Graz, Austria; 2 HEALTH – Institute of Biomedicine and Health Sciences, Joanneum Research, Graz, Austria; 3 Institute of Molecular Biology and Biochemistry, Medical University of Graz, Graz, Austria; Universidade de São Paulo, Brazil

## Abstract

Blood-brain barrier (BBB) impairment in systemic inflammation leads to neuroinflammation. Several factors including cytokines, chemokines and signal transduction molecules are implicated in BBB dysfunction in response to systemic inflammation. Here, we have adopted a novel *in vivo* technique; namely, cerebral open flow microperfusion (cOFM), to perform time-dependent cytokine analysis (TNF-alpha, IL-6 and IL-10) in the frontal cortex of the rat brain in response to a single peripheral administration of lipopolysaccharide (LPS). In parallel, we monitored BBB function using sodium fluorescein as low molecular weight reporter in the cOFM sample. In response to the systemic LPS administration, we observed a rapid increase of TNF-alpha in the serum and brain, which coincides with the BBB disruption. Brain IL-6 and IL-10 synthesis was delayed by approximately 1 h. Our data demonstrate that cOFM can be used to monitor changes in brain cytokine levels and BBB disruption in a rat sepsis model.

## Introduction

Septic encephalopathy, a frequent complication of sepsis, is characterized by blood-brain barrier (BBB) disruption, leucocyte infiltration, up-regulation of aquaporin-4, activation of microglia, astrocytosis, and apoptotic cell death [1], [2]. In rodent sepsis models that utilize intraperitoneal injection of LPS the former symptoms are accompanied by an upregulation of various pro- and anti-inflammatory mediators in brain, comparable to what is found in humans [1], [3]. Consequently, clinical and experimental studies demonstrate that complement activation and the production of inflammatory cytokines could drive BBB disruption, leukocyte recruitment, neuroinflammation, and neuronal cell death [1]. Epidemiological data suggest that about 70% of all patients suffering from systemic inflammatory response syndrome with infection in the intensive care unit develop severe cerebral dysfunction [4]. In a variety of other neurological (inflammatory, infectious, neoplastic and neurodegenerative) diseases, BBB dysfunctions have been described [5]. Brain endothelial barrier disruption in neuroinflammation involves cytokines [6] and these signalling peptides diffuse into interstitial fluid [7] to provide signals to neighbouring cells. Neuropathological and imaging studies demonstrate that loss of BBB integrity precede neuronal damage in many conditions [8]–[10]. Thus, early detection of compromised BBB function could potentially permit early diagnosis and allow testing interventions that will prevent irreversible brain damage.

To investigate the deleterious effects of neuroinflammation on CNS function, direct access to the brain interstitial fluid is needed in order to dynamically assess inflammatory markers. Microdialysis is a widely used technique for studying neurotransmitters, metabolites, inflammatory markers or drugs within the brain [11]–[16]. However, the method suffers several intrinsic drawbacks, including biofouling and clogging of microdialysis-membrane pores [17], [18]. Detection and estimation of cytokines from brain interstitial fluid is therefore difficult because the cytokines cannot easily pass through the membrane pores, leading to low recovery rate, which is further diminished through non-specific adsorption to the outlet tubing and membrane surface. Moreover, tissue analyses for mRNA expression levels of these signalling proteins do not always correlate with the secreted protein levels [19].

To overcome these inherent disadvantages we have recently developed a new membrane-free approach to continuous sampling of brain interstitial fluid: cerebral open flow microperfusion (cOFM). During an earlier study, we demonstrated that BBB disruption due to cOFM probe implantation in the frontal cortex of rats is healed within 15 days of probe implantation. In addition, we showed that cOFM is a promising technique for monitoring transport of substances across the intact BBB [20]. The cOFM probe combines push and -pull perfusion, allowing the perfusate to mix directly with the brain interstitial fluid. Since the technique requires no membrane, it provides access to a wide range of compounds regardless of their size and lipophilicity. Moreover, even minor alterations of BBB permeability can be detected with the help of a suitable low-molecular-weight marker, such as sodium fluorescein (Naf). Of note, the potential contribution of blood (retained in the brain vasculature) to cytokine and chemokine levels in brain [21] can be excluded when applying cOFM.

In the present study, we aimed to characterize the suitability of cOFM for monitoring BBB function and cerebral cytokine production in a rat model. We adopted a well-characterized model for acute systemic inflammation induced by a septic dose of lipopolysaccharide (LPS) [22] to study cytokine synthesis in the brain, and utilized Naf as a marker for BBB permeability. Systemic administration of LPS produces an acute inflammatory response in the brain, which includes BBB disruption [23] and release of cytokines [24], [25]. Using cOFM, we monitored BBB permeability over a prolonged period and investigated the dynamic time profiles of several cytokines in brain and serum.

## Materials and Methods

### Animals

All animal protocols used in this study were approved by the Austrian Ministry of Science and Research Ref.II/10b, Vienna (Permit Number: BMWF-66.010/0003-0024II/3b/2011). A total of 26 adult male Sprague Dawley rats (Harlan Laboratories, Udine, Italy) weighing approximately 350–450 g were used for the study. The animals were housed in a 12 h light/dark cycle with food and water available *ad libitum*. Animals were allowed at least 1 week after transportation to acclimatize to the environment prior to any surgical procedure. Appropriate animal care such as cleaning of cage, bedding etc. was provided by the resource staff at the animal care facility centre (Institute for Biomedical Research, Medical University of Graz, Austria).

### cOFM probes

**Figure 1:**
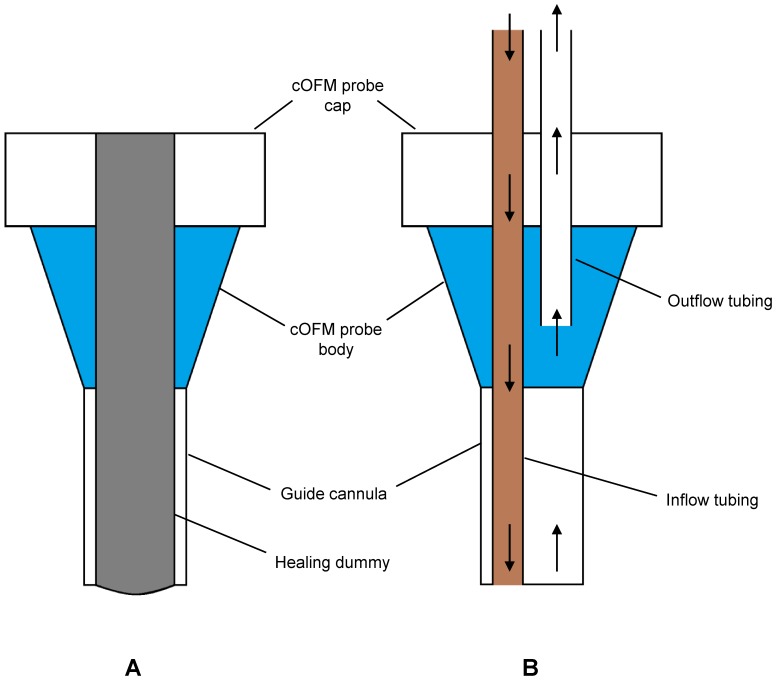
Schematic diagram of cOFM probe with dummy (A) and cOFM probe with inflow/outflow tubing (B).

The cOFM probe consists of a 20 Ga guide tube (fluorinated ethylene propylene – FEP) and two 25 Ga inflow/outflow tubes with low adhesion surface (polytetrafluorethylen – PTFE). After fabrication, the cOFM probes were sterilized by storage in ethanol and then packaged aseptically. During implantation, a healing dummy was placed in the guide tube, allowing tissue regeneration and preventing tissue migration into the guide tube (Fig.1). On the day of perfusion, the healing dummy was replaced by inflow/outflow tubing (Fig.1). cOFM perfusate was pumped into brain tissue and withdrawn at a flow rate of 1 µl/min.

### cOFM perfusate

cOFM standard perfusate was based on the fluid described by McNay and Sherwin [26], composed to match brain extracellular fluid in order to avoid chemical stress for the BBB, but with modifications. Perfusate composition: NaCl (123 mM), MgCl_2_ (0.4 mM; purity ≥98%), CaCl_2_ (0.7 mM; purity ≥93%), KCl (4.3 mM), NaH_2_PO_4_ (1.3 mM), Na_2_HPO_4_ (21 mM) and glucose (4 mM). All reagents were dissolved in sterile water (Aqua bidest, Fresenius Kabi, Austria). In order to remove possible bacterial contamination, the perfusate was filtered through a 0.22 µm sterile filter (Thermo Fisher Scientific, Germany). All subsequent steps were carried out under sterile conditions.

### Surgery and implantation of cOFM probe

For cOFM probe implantation, the rats were anaesthetized with a combination (2∶2∶1) of Fentanyl (0.05 mg/ml; Janssen-Cilag Pharma, Austria), Midazolam (5 mg/ml; Janssen-Cilag Pharma, Austria) and Domitor (0.1 mg/ml, Pfizer Corporation, Austria). A dose of 0.15 ml/100 g of body weight was given subcutaneously. The rats were prepared for surgery by having their heads shaved and being placing in a stereotactic frame (KOPF Instruments, USA). The surgical area of the scalp was disinfected with ethanol (70%). A 2 cm midline incision exposed the skull. cOFM probes were implanted unilaterally in the left frontal cortex, navigating bregma (2 mm left from midline, 0 mm anterior to bregma and 1.5 mm below the dura) under anaesthesia as described above. A hole of 1 mm diameter for the cOFM probe was made in the skull by using a dental drill, taking care not to harm the dura mater. The dura mater was then carefully punctured with fine forceps. Two more such holes were made posteriorly on the skull to fix the anchor screws. The cOFM probe was inserted slowly into the brain with the help of a manipulator arm (David Kopf Instruments, USA) and glued to the anchor screws already placed on the skull with dental cement (i–CEM, Heraeus Kulzer GmbH, Germany) and cured by UV irradiation. The manipulator arm was then carefully detached from the cOFM probe. The surgical procedure was complete within 30 min of initial induction with Isoflurane for each rat. Following surgery, rats were individually housed in specially designed cages under the same conditions. After surgery, the animals received a mixture of Anexate (0.1 mg/ml; Roche GmbH, Austria) and Antisedan (0.5 mg/ml; Pfizer Corporation, Austria) (5: 0.1). For two days after surgery, a daily dose of the antibiotic Claforan (5 mg/100 gm; Sanofi-aventis GmbH, Austria) and Rimadyl (Carprofen 50 mg/ml; Pfizer Corporation, Austria) were injected subcutaneously.

### Systemic inflammation

In order to produce acute systemic inflammation, rats were injected with lipopolysaccharide (LPS; Escherichia coli 0111:B4, Sigma-Aldrich GmbH, Austria) (0.5 ml; 5 mg/kg in normal sterile saline (0.9%)) intraperitoneally after collecting a baseline cOFM sample. Control animals received the same volume of normal sterile saline.

### Determination of BBB permeability in LPS-treated rats

cOFM probe implantation was performed as described above. Fifteen days post surgery the healing dummy was exchanged with inflow and outflow tubing to allow sampling. Perfusion was started one hour after healing-dummy exchange; sampling was started 2.5 hours after healing-dummy exchange, and was continued for seven hours. During sampling, the animals were anesthetized with inhaled isoflurane (1% in 0.5 L/min oxygen, Abbott, Canada). A bolus injection of Naf (11 mg/kg in normal saline) was administered in the femoral vein 1 h after the start of perfusion, followed by a constant infusion of Naf (11 mg/kg/h in normal saline), in order to maintain a steady concentration of Naf in the blood. In the LPS-treated group (n = 6), animals were injected i.p. with LPS (5 mg/kg) after collecting a baseline cOFM sample in order to establish the basal Naf penetration into the brain. The control group (n = 6) received the same volume of normal sterile saline.

### Cytokine measurement

TNF-alpha, IL-6, and IL-10 were measured in the cOFM sample and in serum. cOFM samples were directly stored at −80°C until further use. Blood was collected from the femoral vein and mixed with a commercially available blood-clot enhancer (S-Monovette; Sarstedt, Germany). After 30 min, centrifugation was performed for 10 min at 1000 xg at room temperature. The serum was removed and stored in a low adhesion polypropylene tube at -80°C until further analysis. Cytokine concentration was determined by using a commercially available multiplexed cytokine analysis kit (RCYTO-80K) as recommended by the manufacturer (Millipore GmbH, Austria). The limit of detection of the assays was 4.44 pg/ml for TNF-alpha, 9.8 pg/ml for IL-6 and 5.41 pg/ml for IL-10.

### Statistical analysis

Statistical analysis was performed using PASW statistics 19 (SPSS, USA). A Shapiro-Wilk test was used to examine normal distribution. A Mann-Whitney U Test was performed to determine the level of significance for the BBB permeability study and brain cytokine analysis. We used the one-sample Wilcoxon signed-rank test for serum cytokine analysis. *p* values <0.05 were considered significant.

## Results

### Determination of BBB integrity

**Figure 2:**
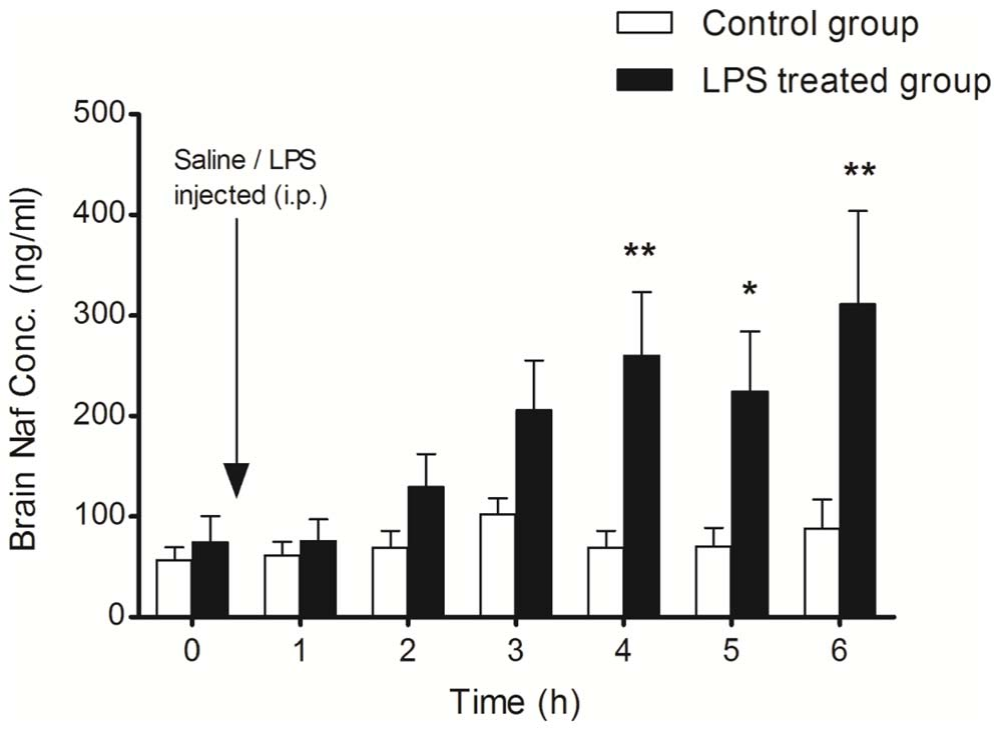
LPS-induced changes in BBB permeability. BBB permeability was measured in terms of Naf accumulation in cOFM samples. Control animals (n = 6) received normal sterile saline (0.5 ml) after the collection of the baseline cOFM sample whereas the LPS-treated group (n = 6) were injected (i.p.) with LPS (5 mg/kg dissolved in 0.5 ml normal sterile saline) after baseline cOFM sample collection. In both groups, a bolus injection of Naf (11 mg/kg dissolved in normal sterile saline) was injected through the femoral vein followed by a continuous infusion of Naf (11 mg/kg/h). Proteins were removed from the cOFM samples by precipitation. Part of the cOFM sample (25 µl) was then mixed with the same volume of acetonitrile at room temperature and Naf concentration was measured. Results are given as mean ± SEM (* p<0.05).

We monitored the alteration of BBB function with time *in vivo* in response to a single systemic LPS bolus by measuring Naf accumulation in the cOFM sample. These analyses revealed that in response to LPS, the Naf concentration in the cOFM sample was higher than baseline after 2 h (Fig. 2). At the end of the experiment (6 h), the Naf concentration in the cOFM sample was 3.5-fold higher in LPS-treated rats than in the saline treated rats (311.5 vs. 87.8 ng/ml).

### Measurement of TNF-alpha in the brain and serum

**Figure 3:**
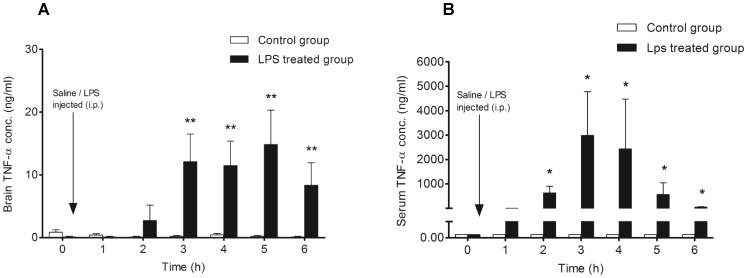
Measurement of TNF-α concentration in the frontal cortex and serum after LPS injection (i.p.). **A**: Control animals (n = 7) received normal sterile saline (0.5 ml; i.p.) after the collection of baseline cOFM samples, whereas the LPS-treated group (n = 7) were injected with LPS (i.p.; 5 mg/kg dissolved in 0.5 ml normal sterile saline) after baseline cOFM sample collection. TNF-alpha concentration in the control animals and in the baseline cOFM sample as well as 1 h after LPS injection was very close to the lower limit of quantification (0.004 ng/ml). **B**: Serum TNF-alpha concentration in control animals and in the baseline sample of the LPS-treated group was below the lower limit of quantification. The serum level of TNF-alpha was found to be significantly elevated from 2 to 6 h after LPS injection. Results are shown as mean ± SEM. (* p<0.05; ** p<0.01).

Intraperitoneal injection of LPS resulted in an increase in TNF-alpha concentration in the frontal cortex after 2 h, with a significant increase 3 to 6 hours after LPS administration (12.08 cf. 8.33 ng/ml; p<0.05) (Fig. 3A). In the control group (saline injected; n = 7), the concentration of TNF-alpha was very low (<0.004 ng/ml) throughout the experiments. On the other hand, a sharp increase in serum TNF-alpha concentration was detected 2 h after LPS administration, reaching the highest concentration at 3 h (246-fold higher than in brain). In contrast to the brain, TNF-alpha concentration in the serum decreased 58-fold (about 2% of maximum serum TNF-alpha concentration) 6 h post LPS administration (Fig. 3B).

### Measurement of IL-6 in the brain and serum

**Figure 4:**
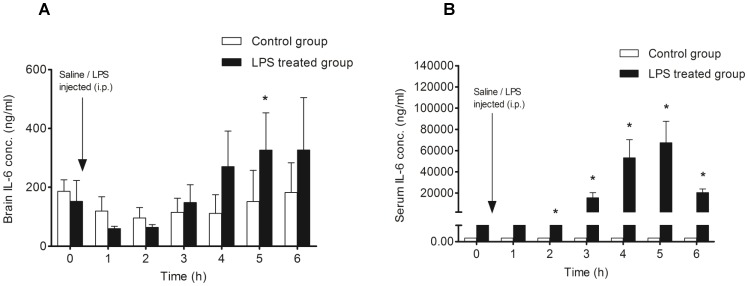
Measurement of IL-6 concentration in the frontal cortex and serum after LPS injection (i.p.). **A**: Control animals (n = 7) received normal sterile saline (i.p.; 0.5 ml) after collection of the baseline cOFM sample, whereas the LPS-treated group (n = 7) were injected with LPS (i.p.; 5 mg/kg dissolved in 0.5 ml normal sterile saline) after baseline cOFM sample collection. **B**: Serum IL-6 concentration in control animals was below the lower limit of quantification (0.009 ng/ml). The serum level of IL-6 was found to be significantly elevated from 2 to 6 h after LPS injection. Results are shown as mean ± SEM. (* p<0.05).

We observed a gradual increase in intracerebral concentration of IL-6 at later (4–6 h) time points post LPS application. The IL-6 concentration was significantly higher (p<0.05) in the cOFM sample than in the control 5 hours after LPS administration (Fig. 4A). A similar time-dependent increase of IL-6 was observed in the serum; however, in contrast to brain, the serum level of IL-6 showed a marked decline at 6 h (Fig. 4B).

### Measurement of IL-10 in brain and serum –

**Figure 5:**
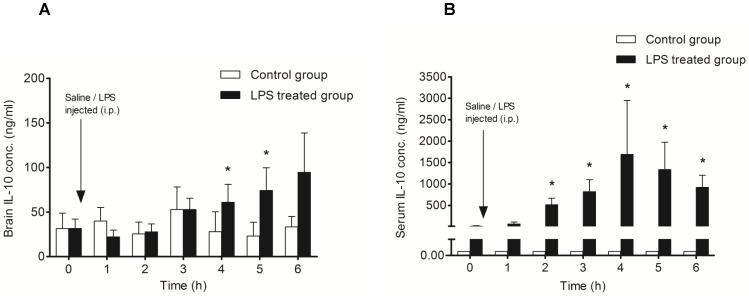
Measurement of IL-10 concentration in the frontal cortex and serum after LPS injection (i.p.). **A**: Control animals (n = 7) received normal sterile saline (i.p.; 0.5 ml) after collection of the baseline cOFM sample, whereas the LPS-treated group (n = 7) were injected with LPS (i.p.; 5 mg/kg dissolved in 0.5 ml normal sterile saline) after baseline cOFM sample collection. **B**: Serum IL-10 concentration in control animals was below the lower limit of quantification (0.005 ng/ml). The serum level of IL-10 was found significantly elevated from 2 to 6 h after LPS injection. Results are shown as mean ± SEM. (* p<0.05).

The intracerebral concentration of IL-10 was significantly higher (p<0.05) at 4 and 5 hours post LPS injection, showing a constant increase with time (Fig. 5A). In contrast, elevated IL-10 levels were detected in the serum after 2 hours, with a peak at 4 hours followed by a gradual decline (Fig. 5B). These results reveal dynamic changes in the levels of intracerebral cytokines in an early phase of neuroinflammation triggered by acute systemic inflammation.

## Discussion

BBB hyper-permeability may lead to neurotoxicity and is implicated in the pathophysiology of neuroinflammation [5]. Data obtained during the present study provide *in vivo* evidence that cOFM is a robust experimental tool to time-dependently monitor BBB function and the production of cytokines in brains of living animals. Using this newly established technology, we found that peripheral LPS administration induced severe BBB dysfunction (monitored by Naf accumulation in the cOFM samples) that was accompanied by increased production of pro- (TNF-alpha and IL-6) and anti-inflammatory (IL-10) cytokines in brain interstitial fluid. Furthermore we showed that TNF-alpha concentrations in brain extracellular fluid and serum increased rapidly, whereas brain IL-6 and IL-10 production lagged behind approximately 1 h.

In response to a single injection of LPS, we observed significant breakdown of BBB function as indicated by increased Naf concentrations in the cOFM samples. Rosenberg et al. demonstrated that endotoxin-induced vascular endothelial permeability is due to the release of cytokines, free radicals, matrix-metalloproteinases, nitric oxide and products of the arachidonic acid cascade [27], [28]. Although LPS only minimally penetrates the intact BBB [29] signal transduction via Toll-like receptors on brain endothelial cells [30] can elicit downstream events that are implicated in the disruption of the tight-junction architecture. Upon activation, RhoA, nuclear factor kappa B, phosphoinositide 3-kinase, myosin light chain kinase, protein kinase C or the MAPK members have been shown to participate in these pathophysiological processes [31]–[34]. Ultimately output of these signalling cascades can impact on barrier function by directly affecting tight-junction architecture proteins or by mediating indirect effects on junctional complexes via cytoskeletal arrangements [35].

In addition to the structural barrier that is formed by brain microvascular endothelial cells, the BBB also fulfils an important biochemical barrier function. This is achieved through polarized expression of highly specific transport systems on brain endothelial cells [36]. Many of these specific transport systems show altered function under inflammatory conditions suggesting that inflammation can affect disease progression via the BBB. Along the same line, transport of insulin, leptin, amyloid beta or TNF-alpha is affected by peripheral LPS administration [37]–[40]. Although the underlying mechanisms are not completely understood, cytokines and chemokines are presumed to play a central role in these processes.

During the present study, we monitored LPS-induced BBB disruption in individual animals over a period of 6 h, and measured the kinetics of TNF-alpha, IL-6, and IL-10 production in serum and brain by cOFM after a single LPS injection. In this regard, it is important to note that cytokine profiles that develop during the inflammatory response heavily depend on the LPS injection paradigm [21]. In terms of time dependency we found that the inflammatory response in brain resolves more slowly than the peripheral response, which is in line with previous observations [21], [41]. This might be due to different Toll-like receptor signaling in the brain [42] and the periphery [43] as well as minimal penetration of LPS across the BBB [29].

The term neuroinflammation describes a broad range of immune reactions in the CNS with multiple stimuli and cell populations involved [44]. The latter include endothelial cells at the BBB, vascular pericytes, microglia, astrocytes, or neurons. The BBB is capable of cytokine transcytosis [40], transmits cytokine-elicited signals, and is capable of endogenous cytokine synthesis [45]. Microglia respond to exogenous stimuli by pronounced alterations of morphology, transforming from a ramified to an amoeboid phenotype and release cytokines and neurotoxic mediators that contribute to long-term neurodegeneration [46]. Astrocytes are able to release pro-inflammatory mediators upon stimulation and thereby contributing to neurodegeneration [47]. The question which of these cell populations is responsible for cytokine production was not addressed experimentally during the present study. However, in light of the relatively short time course investigated here (up to 6 h) it might be reasonable to assume that cells at the neurovascular unit are the first to respond to peripheral cytokines. This assumption would be in line with findings reported by Denes et al. [48]: These authors reported (that in addition to parenchymal cells with microglial morphology) IL-1 positive perivascular cells were enriched around dilated blood vessels in mouse brain in response to LPS or ovalbumin.

Under normal physiological conditions, the basal level of TNF-alpha remains low, but TNF-alpha concentration increases in acute inflammation, trauma and autoimmune diseases [49]. In the LPS-treated group, brain TNF-alpha concentrations started to increase 2 h post LPS injection (Fig. 3), which coincides with the time course of BBB dysfunction (Fig. 2), indicating a relationship between TNF-alpha production and BBB function. This is supported by a previous report demonstrating that BBB disruption in sepsis is mediated by TNF-alpha signaling through TNFR1 [2]. In addition it was shown that TNF-alpha is responsible for increased BBB permeability in E.coli induced meningitis [50]. In an MPTP-induced mouse model of Parkinson disease it was shown that TNF-alpha knockout also significantly attenuated BBB dysfunction [51]. However, it is noteworthy that LPS from different sources can affects BBB function differentially, despite equally increased serum TNF-alpha levels [52]. In the present study, we noticed that the increase in serum TNF-alpha was transient and decreased dramatically at 6 h. In contrast, brain TNF-alpha remained elevated throughout the experiment, indicating slower resolution of the inflammatory response in the brain, findings that are in line with results obtained in LPS mouse models [21], [41].

In addition, we observed delayed intracerebral production of IL-6 in response to peripheral LPS challenge (Fig. 4). This could indicate indirect stimulation of intracerebral IL-6 production via TNF-alpha. Results from a chemokine network analysis in an LPS mouse model are in support of this notion [21]. Along the same line, an earlier study demonstrated that LPS induced production of IL-6 in brain was mediated by TNF [53]. It was also shown that TNF-alpha and IL-1 induce release of IL-6 by lymphocytes, glial cells, and neurons [54]. Moreover, immunohistochemical studies on brain sections revealed an early appearance of TNF-alpha and IL-1beta positive cells followed by IL-6 positive cells in excitotoxic brain lesion [55]. Therefore, it is plausible that a synergistic action between TNF-alpha and IL-6 contributes to a self-propelling neuroinflammatory environment.

The decrease in concentration of proinflammatory cytokines in the serum after LPS administration was probably due to the gradual increase in anti-inflammatory IL-10 (Fig. 5). This fact is supported by an earlier observation that IL-10 can suppress synthesis of proinflammatory cytokines, such as TNF-alpha, IL-6, and IL-1 by targeting circulating immune cells, liver, and spleen [56], [57], and can down-regulate receptors for proinflammatory cytokines [58]. We detected a slight increase in serum IL-10 as early as 1 h after LPS injection. In contrast, we found that the brain concentration of IL-10 gradually increased and remained significantly elevated until the end of the experiment. Generally, an increase in anti-inflammatory cytokines levels results in the subsequent decline of proinflammatory cytokines with time, as we documented in this study. Therefore, it remains unclear why intracerebral TNF-alpha and IL-6 levels remained high despite the higher intracerebral concentration of IL-10. It was previously reported that a single intraperitoneal injection of LPS provoked a rapid increase of TNF-alpha that lasted for months [41]. Therefore, it seems that the production of anti-inflammatory cytokines such as IL-10 in brain is insufficient to attenuate TNF-alpha production. In addition, it was shown that IL-10 mRNA in the cortex had returned to baseline levels 8 hours after a single LPS injection [59]. Moreover the biological half-life of IL-10 is very short [56], [60].

In summary, we have performed a time-dependent cytokine analysis (TNF-alpha, IL-6 and IL-10) in the frontal cortex of the rat brain in response to a single peripheral administration of lipopolysaccharide (LPS) by using the newly developed technique, cOFM. We monitored BBB function by using sodium fluorescein as low-molecular-weight reporter in the cOFM sample. Our data demonstrate that cOFM is well suited for detecting alterations of BBB permeability in living rats, and monitoring changes in brain cytokine levels in response to a peripheral endotoxin challenge.
